# Dose-Response Relationship of Neuromuscular Training for Injury Prevention in Youth Athletes: A Meta-Analysis

**DOI:** 10.3389/fphys.2017.00920

**Published:** 2017-11-14

**Authors:** Simon Steib, Anna L. Rahlf, Klaus Pfeifer, Astrid Zech

**Affiliations:** ^1^Department of Sport Science and Sport, Friedrich-Alexander-University Erlangen-Nürnberg, Erlangen, Germany; ^2^Institute of Sport Science, Friedrich-Schiller-University of Jena, Jena, Germany

**Keywords:** exercise, sensorimotor training, balance training, injuries, children, youth, adolescent, team sports

## Abstract

**Background:** Youth athletes with intensive sports participation are at an increased risk of sustaining injuries. Neuromuscular training programs reduce sports-related injury risk in this population, however, the dose-response relationship is largely unknown. Thus, the aim of this meta-analysis was to identify the optimal frequency, volume, duration, and period of neuromuscular training to prevent injuries in youth athletes.

**Methods:** Computerized database searches (PubMed, Scopus, SPORTDiscus, The Cochrane Library, PEDro) were conducted in January 2017, with search terms related to youth sports, neuromuscular training, and injury prevention. Eligible trials (i) evaluated a neuromuscular training program; (ii) included youth athletes of 21 years or younger; (iii) had an analytical design (RCTs, quasi-experimental, cohort studies); (iv) contained original data; (v) and provided injury data. Two reviewers independently extracted data and assessed quality of eligible studies. Injury rate ratios (IRRs) for lower extremity injuries were pooled meta-analytically, and moderator analyses examined the effect of training frequency, duration, volume, and period.

**Results:** Data from 16 trials yielded an overall risk reduction of 42% with neuromuscular training (IRR = 0.58, 95%CI 0.47–0.72). Training frequencies of two (IRR = 0.50; 95%CI 0.29–0.86) or three times (IRR = 0.40; 95%CI 0.31–0.53) per week revealed the largest risk reduction, and a weekly training volume of more than 30 min tended to be more effective compared to lower volumes. Programs with 10–15 min (IRR = 0.55; 95%CI 0.42–0.72) session duration produced effects comparable to those with longer session duration (IRR = 0.60; 95%CI 0.46–0.76). Interventions lasting more than 6 months were not superior to shorter programs.

**Conclusion:** This meta-analysis revealed that NMT performed in short bouts of 10–15 min, two to three times per week, with a weekly training volume of 30–60 min had the largest preventive effect for lower extremity injuries in youth athletes. These effects can be achieved within 20–60 sessions and training periods of <6 months. The present results are derived from a relatively small number of studies with heterogeneous methodological quality and should be treated with caution.

The study was a priori registered at PROSPERO (CRD42016053473).

## Introduction

The high participation rates as well as a growing specialization and professionalization of sports in young ages entail multiple benefits. However, this comes at the expense of an increased risk of injury and illness. The sport-related injury risk of youth athletes has been demonstrated in a variety of age ranges and sport activities (Pickett et al., 2005; Emery and Tyreman, 2009), with incidence rates of up to 34.4/1,000 h of sport exposure reported in young male ice hockey players for instance (Caine et al., 2008). These data emphasize the urgent need for developing effective strategies to prevent injuries. Hence, a growing number of injury prevention programs have been developed in recent years, with the majority containing multiple exercise components addressing neuromuscular performance (Emery et al., 2015). The positive effects of neuromuscular training (NMT) programs on the incidence of injuries in adults are well established (Hübscher et al., 2010; Lauersen et al., 2014; Schiftan et al., 2015; al Attar et al., 2016). A recent meta-analysis found that a soccer-specific NMT program reduced injury rates by 20–50% (al Attar et al., 2016). Regarding ankle injuries, neuromuscular multi-intervention, and proprioceptive programs have been found to decrease risk by 35–50% in sporting adult populations (Hübscher et al., 2010; Schiftan et al., 2015). Similar effects have also been reported for youth athletes. Two meta-analyses have demonstrated a risk reduction for lower extremity injuries of around 25–35% (Emery et al., 2015; Soomro et al., 2016).

While the preventive effects of neuromuscular interventions in youth athletes are indisputable, little is known with respect to their optimal dose. The investigated programs vary substantially with respect to training content and individual dosage parameters. Neuromuscular injury prevention programs for youth athletes have been examined in various sports, including basketball (Hewett et al., 1999; McGuine and Keene, 2006; Emery et al., 2007; LaBella et al., 2011), handball (Wedderkopp et al., 1999; Olsen et al., 2005), soccer (Hewett et al., 1999; Heidt et al., 2000; Malliou et al., 2004; Mandelbaum et al., 2005; Soligard et al., 2008; Steffen et al., 2008), and volleyball (Hewett et al., 1999; Heidt et al., 2000). Besides sport-specific contents, these programs typically either include multiple components, or focus on balance exercises (Hübscher et al., 2010; Zech et al., 2010). Frequent contents of multi-intervention programs include strength, balance, flexibility, plyometric, speed, and agility exercises (Hübscher et al., 2010), thereby focusing on neuromuscular control and active joint stabilization. Importantly, parameters such as the duration and volume of single training sessions, the training frequency, the intervention volume or training period vary substantially between individual studies (Soomro et al., 2016). Hence, it is difficult to infer the most effective training prescription based on findings from individual studies. A better understanding of dose-response relationships is a fundamental basis for designing well-tailored, population-specific exercise programs.

At present, prospective studies on the analysis of the dose-response relationship in NMT programs are lacking. In adult athletes, preliminary evidence has suggested that a session duration of at least 10 min, and a training frequency of more than once a week for at least 3 months is necessary in order to prevent injuries (Hübscher et al., 2010). In addition, the optimal dose to prevent anterior cruciate ligament injuries in female athletes should include training for at least twice a week, with a minimum of 20 min per session (Sugimoto et al., 2014). Two recent meta-analyses which investigated dosage-effects of balance training, a key component of NMT programs, reported the largest improvements in neuromuscular outcomes with training frequencies of three times a week, session durations of 11–15 min, and training periods of ~12 weeks (Zech et al., 2010; Lesinski et al., 2015). In youth athletes on the other hand, information on the optimal dose of NMT is scarce. However, such information is particularly relevant in this population, considering the biological and anthropometric inter-individual differences caused by the maturational status. The little data available on dose-response relationships has suggested that training periods of more than 8 months have comparable preventive effects compared to shorter periods (Soomro et al., 2016). From a practical point of view, a deeper understanding of the best training dosage is crucial for tailoring training parameters to the specific population, and would increase coaches' and athletes' confidence in applying NMT programs (van Tiggelen et al., 2008; Zech and Wellmann, 2017).

Taken together, although NMT programs have demonstrated preventive effects in youth athletic populations, no consistent recommendations can be inferred from the current literature with respect to the duration, frequency, volume and training period of such programs. Establishing the minimal and optimal effective dose would not only help practitioners in designing tailored programs, but could also increase coaches' and athletes' compliance to such interventions (van Tiggelen et al., 2008; Zech and Wellmann, 2017). This is particularly relevant in youth athletes, where differences in maturational status can cause large inter-individual variation in anthropometrics and neuromuscular performance. Consequently, this systematic review and meta-analysis aimed to investigate dose-response relationships of NMT programs to prevent lower extremity injuries in adolescent athletes. Specifically, the optimal training frequency, session duration, training volume, and intervention period were targeted to provide recommendations for sports practice.

## Methods

This systematic review and meta-analysis was preregistered (registration number: CRD42016053473) at the international prospective register of systematic reviews (PROSPERO). The registration protocol is accessible at http://www.crd.york.ac.uk/PROSPERO/display_record.asp?ID=CRD42016053473.

### Search strategy

A systematic computerized database search was conducted in five databases (PubMed, Scopus, SPORTDiscus, The Cochrane Library, PEDro) from their inception up until January 12, 2017. Articles in English and published in peer-reviewed journals were considered. We developed a systematic search strategy by clustering key terms according to the PICOS (Patient/Problem, Intervention, Control/Comparison, Outcome, Study design) strategy. Selected key words related to youth sports, neuromuscular training, and injury prevention were connected using Boolean terms. A detailed list of the exact terms and search strategies used is provided in the Supplementary Material. In addition to electronic database searching, the reference lists of articles were searched during full text screening in an effort to obtain additional eligible studies.

### Selection criteria

Based on the PICOS strategy, the following criteria had to be fulfilled in order for studies to be considered in this meta-analysis: (i) the study population consisted of youths of 21 years or younger (Malina et al., 2015), participating in structured/organized sport programs on a competitive level (P); (ii) a neuromuscular training program (including components such as balance, agility, strength, neuromuscular control) was evaluated with no co-interventions (e.g., education) provided (I); (iii) the study contained a control arm either performing usual practice routine or sham exercises without specific focus on neuromuscular control (C); (iv) data for at least one outcome of lower extremity sports injury was provided (O); and (v) an analytical design was used (RCTs, quasi-experimental trials, cohort studies) (S). Studies without original data (review articles) or without obtainable data for meta-analysis were excluded.

### Risk of bias assessment

We analyzed risk of bias of included studies using the PEDro scale (Maher et al., 2003). This scale consists of eleven items, addressing internal validity (8 items), interpretability (2 items), and external validity (1 item). A point was scored for each item clearly fulfilling the criterion, allowing a maximal score of 11 points. Two reviewers (SST, ALR) independently performed the quality rating. Disagreements between ratings were discussed and solved via consensus. This process was piloted on three studies not included in the review before actual quality rating was performed.

### Data extraction

Two researchers (SST, ALR) extracted predefined study characteristics from publications and collected the information in tabular form. These characteristics included authors, publication year, study design, participants (age, gender, sports, expertise level, sample size), interventions (types of exercises, training period, training frequency, number of sessions, and session duration for experimental and control groups, respectively), and results (type of injury, injury incidence by type/ location, player exposures).

### Outcome measures

Data was extracted for lower extremity (LE) injury, including any form of muscular, ligamentous or bony injuries (traumatic or overuse). If available, the total number of LE injuries was used for meta-analysis. In cases where studies only reported knee or ankle injuries, this data was used accordingly.

NMT dosage was divided into the following components: training session duration and frequency, weekly volume, and total intervention volume and period.

– *Session duration:* The time (minutes) spent for one NMT session.
– *Training frequency:* The weekly number of NMT sessions.
– *Weekly training volume:* The time (minutes) per week spent for NMT (training frequency x session duration).
– *Intervention volume:* The total number of training sessions, equaling the sum of all sessions throughout the intervention period.
– *Intervention period:* The total intervention duration in weeks.

For moderator analyses, further subgroups were formed within each variable: *session duration* was categorized into low (10–15 min), medium (20–30 min), and high (>30 min); *training frequency* was clustered into 1x, 2x, 3x, and >3x per week; *weekly volume* was categorized as low (<30 min), medium (30–60 min), and high (>60); *intervention volume* was clustered into low (<30 sessions), moderate (30–60 sessions), and high (>90 sessions); and *intervention period* was separated into short term (≤6 months) and long-term (>6 months).

### Statistical analysis

#### Meta-analysis

All analyses were performed using the Cochrane review manager (version 5.3.5, The Cochrane Collaboration, Copenhagen). Injury rate ratios (IRRs) with corresponding 95% confidence intervals (CI) were calculated representing an effect estimate for each included study: IRR = (number of injuries in NMT group/player exposures)/(number of injuries in control group/player exposures). In cases where player exposure hours were not available, IRRs were calculated using the players' number of practice and game exposures. The IRR resembles a ratio of the within-group (NMT, control) injury incidence rates. Consequently, a value smaller than 1 indicates an injury risk reduction in favor of NMT, and the closer the value to 0, the larger is the effect. Both cluster RCTs and cohort studies were included in this meta-analysis, and sensitivity analyses indicated that no systematic difference in effect sizes existed between these study designs (*I*^2^ = 0%; *Q* = 0.14; *p* = 0.71).

As significant heterogeneity in individual studies' IRRs was present (*I*^2^ = 71%, *Q* = 55.77; *p* < 0.0001), the assumption of a unified true intervention effect was dismissed. Consequently, a random effect model (inverse-variance) was used for weighting individual studies and estimating the overall pooled effect size (IRR). Z statistics and respective *P*-values were calculated to assess whether this effect was statistically significant. Heterogeneity between studies' IRRs were observed using chi-squared tests, and *I*^2^ values were calculated to quantify the proportion (in %) of observed variance.

Sensitivity analyses were performed for the influence of *study design* (RCT vs. cohort study) and *study quality* on the overall effect, in order to detect potential bias from studies with lower levels of internal validity.

#### Moderator analysis

Moderator analyses were performed in order to examine whether specific dosage features would have a stronger effect on injury risk reduction. The following moderators were examined: (i) training frequency; (ii) training session duration; (iii) weekly training volume; (iv) total number of training sessions; (v) intervention period.

For each moderator, subgroups were defined as described above (data extraction). A pooled effect estimate was then calculated for each subgroup containing at least two studies, and differences between subgroups were tested by assessing heterogeneity across subgroup effects using chi-squared tests. Besides statistical comparison, we descriptively compared subgroups IRRs, considering a ≥10% difference as meaningful (Soomro et al., 2016). Meta-regression was not performed due to the limited number of studies and the lack of precision in the continuous data (e.g., training session duration data reported resembled the prescribed duration, rather than the actually performed and precisely measured time).

## Results

### Trial flow

**Figure 1:**
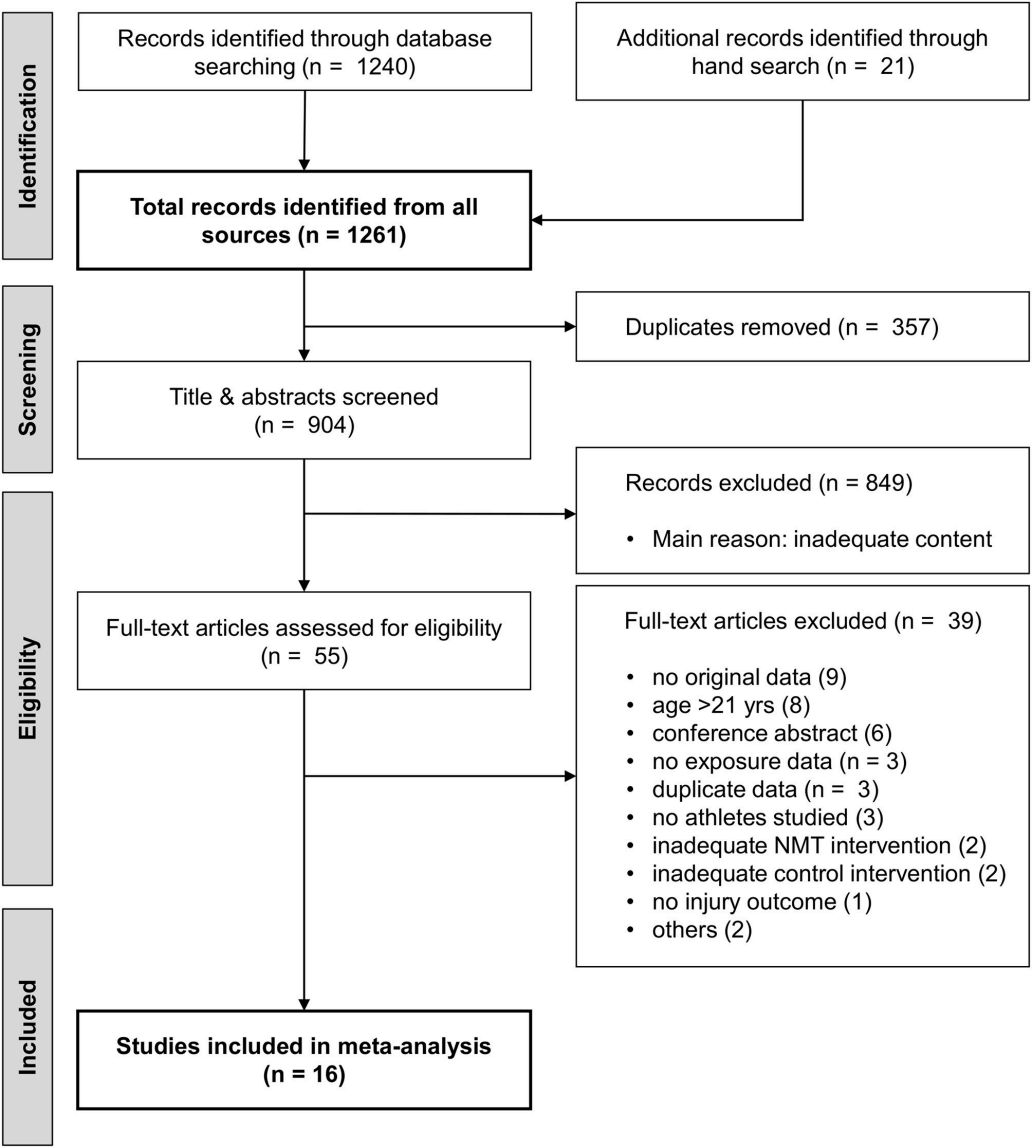
PRISMA flow chart.

Our search strategy identified a total of 1261 records (Figure 1). We screened titles and abstracts from 904 articles after duplicate removal. From these, 849 were discarded and full texts obtained from the remaining 55 articles. After full text screening, another 39 articles were excluded, mostly due to lack of original data or inadequate study population. Consequently, 16 trials were included in the final meta-analysis.

### Study characteristics

Table 1 shows the characteristics of the included studies. The sample size varies substantially, from 54 (Cumps et al., 2007) up to 4,546 (Walden et al., 2012). In six studies, both male and female athletes were examined (Hewett et al., 1999; Olsen et al., 2005; McGuine and Keene, 2006; Cumps et al., 2007; Emery et al., 2007; Emery and Meeuwisse, 2010), while two studies focused on male athletes (Longo et al., 2012; Owoeye et al., 2014), and seven trials on females only (Wedderkopp et al., 1999; Mandelbaum et al., 2005; Pfeiffer et al., 2006; Soligard et al., 2008; Steffen et al., 2008; LaBella et al., 2011; Walden et al., 2012). The mean age of the participants varied between 14 (Walden et al., 2012) and 17 years (Steffen et al., 2008; Owoeye et al., 2014), and age groups typically ranged from 12 to 18 years. With respect to players' competitive level, 12 studies focused on sub-elite athletes organized in clubs (Mandelbaum et al., 2005; Olsen et al., 2005; Soligard et al., 2008; Emery and Meeuwisse, 2010; Walden et al., 2012) or high-school sports (Hewett et al., 1999; McGuine and Keene, 2006; Pfeiffer et al., 2006; Emery et al., 2007; McHugh et al., 2007; Steffen et al., 2008; LaBella et al., 2011). Three studies investigated elite players (Cumps et al., 2007; Longo et al., 2012; Owoeye et al., 2014), and one study included a mixed sample (Wedderkopp et al., 1999). All trials studied team sport athletes, with soccer (Mandelbaum et al., 2005; Pfeiffer et al., 2006; Soligard et al., 2008; Steffen et al., 2008; Emery and Meeuwisse, 2010; Walden et al., 2012; Owoeye et al., 2014), and basketball (Cumps et al., 2007; Emery et al., 2007; Longo et al., 2012) being the most common. The NMT programs typically consisted of either multiple components (generally strength, balance, and agility) or balance exercises only. The most commonly investigated standardized multi-component programs were FIFA “the 11” (Steffen et al., 2008) or FIFA “11+” (Soligard et al., 2008; Longo et al., 2012; Owoeye et al., 2014). Balance training only was used in six studies (Wedderkopp et al., 1999; McGuine and Keene, 2006; Cumps et al., 2007; Emery et al., 2007; McHugh et al., 2007).

**Table 1:** Characteristics of included studies.

**Author, year**	**Sample size (n)^a^**	**Age (years), mean ± SD^b^^,^^e^**	**Sport**	**Level^d^**	**Intervention group**	**Control group**	**Period (weeks)**	**Duration (minutes)**	**Frequency (per week)**	**Primary outcome**	**Number of injuries^b^**
Cumps et al., 2007	F:17, M:37	IG:17.7 ± 3.9, CG:18 ± 2.7	Basketball	Elite	Balance training intervention (warm-up including basketball-skills on balance semi-globes, progressive difficulty)	Normal routine	22	5–10	3	Ankle injury	IG:5, CG:10
Emery et al., 2007	F:456, M:464	12–18 (range)	Basketball	Sub-elite	Balance training (10 min warm-up routine including aerobic and stretching and 5 min sport-specific balance training warm-up + 20 min home training on wobble board)	Standardized warm-up (specified by research team)	18	15–20	5	Any LE injury, ankle injury	IG:106, CG:111
Emery and Meeuwisse, 2010	F/M:1018	13–18 (range)	Indoor soccer	Sub-elite	Soccer-specific neuromuscular training program (5 min aerobic and stretching and 10 min neuromuscular components including strength, agility, and balance + 15 min home-based balance training on wobble board)	Standardized warm-up (specified by research team)	20	15	No data	Any LE injury, knee injury, ankle injury	IG:42, CG:60
Hewett et al., 1999	F:829, M:434	No data	Soccer, volleyball, basketball	Sub-elite	Pre-season neuromuscular training program (jump technique and performance, stretching and weight training)	No preseason neuromuscular training program	6	60–90	3	Knee injury	IG:2, CG:10
LaBella et al., 2011	F:1,558	IG:16.2 ± 1.5, CG:16.2 ± 1.1	Basketball, soccer	Sub-elite	Neuromuscular warm-up training program (progressive strengthening, plyometric, balance, and agility exercises)	Usual warm-up	9–18^c^	20	3	Any LE injury, knee injury, ankle injury	IG:50, CG:96
Longo et al., 2012	M:221	IG:13.5 ± 1.2, CG:15.2 ± 4.6 11–24 (range)	Basketball	Elite	Neuromuscular warm-up training program—FIFA 11+ (running, strength, balance, jumping exercises, and agility)	Usual warm-up	36	20	3–4^f^	Any LE injury, knee injury, ankle injury	IG:10, CG:11
Mandelbaum et al., 2005	Year 1: F:2,946, year 2: F:2,757	14–18 (range)	Soccer	Sub-elite	Prevent Injury and Enhance Performance (PEP) program (warm-up activities, stretching techniques, strengthening exercises, plyometric activities, soccer-specific agility drills)	Usual warm-up	16	20	2.18	ACL injury	Year 1: IG:2, CG:32; year 2: IG:4, CG:35
McGuine and Keene, 2006	F:523, M:242	IG:16.4 ± 1.2, CG:16.6 ± 1.1	Basketball, soccer	Sub-elite	Balance training program (single- and double-limb balance training on balance board, including 5 phases)	No balance training program	No data	10	3 (preseason 5)	Ankle injury	IG:23, CG:39
McHugh et al., 2007	125	15–18 (range)	American football	Sub-elite	Balance training intervention (single-limb balance training on a foam stability pad)	No control group	13	10	2 (preseason 5)	Ankle injury	IG:20, CG:21
Olsen et al., 2005	F:1,586, M:251	IG:16.3 ± 0.6, CG:16.2 ± 0.6, 15–17 (range)	Handball	Sub- elite	Neuromuscular warm-up training program (exercises with ball, wobble board and balance mat to improve technique, balance and strength)	Usual warm-up	32	15–20	1	Any LE injury, knee injury, ankle injury	IG:66, CG:115
Owoeye et al., 2014	M:416	IG:17.8 ± 0.9, CG:17.5 ± 1.1, 14–19 (range)	Soccer	Elite	Neuromuscular warm-up training program—FIFA 11+ (running, strength, balance, jumping exercises and agility)	Usual warm-up	24	20	2	Any LE injury, knee injury, ankle injury	IG:26, CG:76
Pfeiffer et al., 2006	F:1,439	No data	Soccer	Sub-elite	Knee Ligament Injury Prevention (KLIP) program (plyometric and agility exercises)	No KLIP program	24	20	2	ACL injury	IG:3, CG:3
Soligard et al., 2008	F:1,892	15.4 ± 0.7, 13–17 (range)	Soccer	Sub-elite	Neuromuscular warm-up training program- FIFA 11+ (running, strength, balance, jumping exercises and agility)	Usual warm-up	32	20	2-6^f^	Any LE injury, knee injury, ankle injury	IG:121, CG:141
Steffen et al., 2008	F:2,100	17.1 ± 0.8, 13–18 (range)	Soccer	Sub-elite	Neuromuscular warm-up training program—FIFA “the 11”(core stability, balance, eccentric hamstrings strength and dynamic stabilization)	Usual warm-up and training	32	20	1	Any LE injury, knee injury, ankle injury	IG:181, CG:173
Walden et al., 2012	F:4,564	IG:14.0 ± 1.2, CG:14.1 ± 1.2, 12–17 (range)	Soccer	Sub-elite	Neuromuscular warm-up training program (focus on knee control and core stability in six exercises)	Usual training and play	28	20	2	Knee injury	IG:49, CG:47
Wedderkopp et al., 1999	F:237	16–18 (range)	Handball	Mix	Balance training + functional activities for the upper and lower extremities (ankle disk training, warm-up and training of all muscle groups)	Usual practice and play	40	10–15	3	Any LE injury, knee injury, ankle injury	IG:10, CG:37

a *If data available distributed by sex (F, Female; M, Male)*.

b *If data available distributed in IG: intervention group and CG: control group*.

c *9–12 weeks in soccer, 15–18 weeks in basketball*.

d *Sub-elite and elite organized in club or high-school sports, mix includes different levels*.

e *For missing mean ± SD the range is specified*.

f *Depending on the number of training sessions per week; ACL, Anterior Cruciate Ligament*.

The training parameters reported in the included studies were as follows: The duration of NMT sessions varied from 5 to 10 (Cumps et al., 2007) up to 60–90 min (Hewett et al., 1999), but the majority of trials (*N* = 12) implemented sessions of 15–20 min length (Wedderkopp et al., 1999; Mandelbaum et al., 2005; Olsen et al., 2005; Pfeiffer et al., 2006; Emery et al., 2007; Soligard et al., 2008; Steffen et al., 2008; Emery and Meeuwisse, 2010; LaBella et al., 2011; Longo et al., 2012; Walden et al., 2012; Owoeye et al., 2014). Training frequencies of two and three times a week were most common, however, three studies reported one (Olsen et al., 2005; Steffen et al., 2008) or five (Emery et al., 2007) weekly sessions. The intervention period varied between 6 (Hewett et al., 1999) and 40 weeks (Wedderkopp et al., 1999), and the total number of scheduled training sessions ranged from 18 (Hewett et al., 1999) to 140 sessions (Wedderkopp et al., 1999).

### Methodological quality

Table 2 presents the methodological quality assessment of included studies. The majority of studies were of moderate (PEDro score 5–7; *N* = 5) or high (PEDro score ≥ 8; *N* = 5) methodological quality. Six studies presented low methodological quality (PEDro score 1–3). Statistical between-group comparisons were reported in all trials, and all but one study provided point and variability measures for key outcomes. Other criteria that were fulfilled in the majority of studies include random group allocation (*N* = 11), intention-to-treat analysis (*N* = 10), specification of eligibility criteria (*N* = 10), and allocation concealment (*N* = 8). Similarity of experimental groups at baseline was ensured in only six trials. Similarly, only six trials reported assessor blinding and follow-up data from more than 85% of participants.

**Table 2:** Quality assessment (PEDro) of included studies.

**First author, year**	**Eligibility criteria specified**	**Randomization**	**Concealed allocation**	**Baseline comparability**	**Patients blinded**	**Care provider blinded**	**Assessor blinded**	**Adequate follow up**	**Intention-to-treat**	**Between-group comparisons**	**Point estimates and variability**	**Score**
Cumps et al., 2007	–	–	–	–	–	–	–	✓	–	✓	✓	3
Emery et al., 2007	✓	✓	✓	✓	–	–	✓	✓	✓	✓	✓	9
Emery and Meeuwisse, 2010	✓	✓	✓	✓	–	–	✓	–	✓	✓	✓	8
Hewett et al., 1999	–	–	–	–	–	–	–	–	–	✓	–	1
LaBella et al., 2011	✓	✓	–	–	–	–	–	✓	✓	✓	✓	6
Longo et al., 2012	✓	✓	✓	–	–	–	✓	✓	✓	✓	✓	8
Mandelbaum et al., 2005	✓	–	–	–	–	–	–	–	–	✓	✓	3
McGuine and Keene, 2006	✓	✓	–	✓	–	–	–	–	✓	✓	✓	6
McHugh et al., 2007	–	–	–	–	–	–	–	–	–	✓	✓	2
Olsen et al., 2005	✓	✓	✓	✓	–	–	✓	✓	✓	✓	✓	9
Owoeye et al., 2014	–	✓	✓	✓	–	–	–	✓	✓	✓	✓	7
Pfeiffer et al., 2006	–	–	–	–	–	–	–	–	–	✓	✓	2
Soligard et al., 2008	✓	✓	✓	–	–	–	✓	–	✓	✓	✓	7
Steffen et al., 2008	✓	✓	✓	–	–	–	✓	✓	✓	✓	✓	8
Walden et al., 2012	✓	✓	✓	✓	–	–	–	–	✓	✓	✓	7
Wedderkopp et al., 1999	–	✓	–	–	–	–	–	–	–	✓	✓	3

*✓ Criterion fulfilled; – Criterion not fulfilled*.

**Figure 2:**
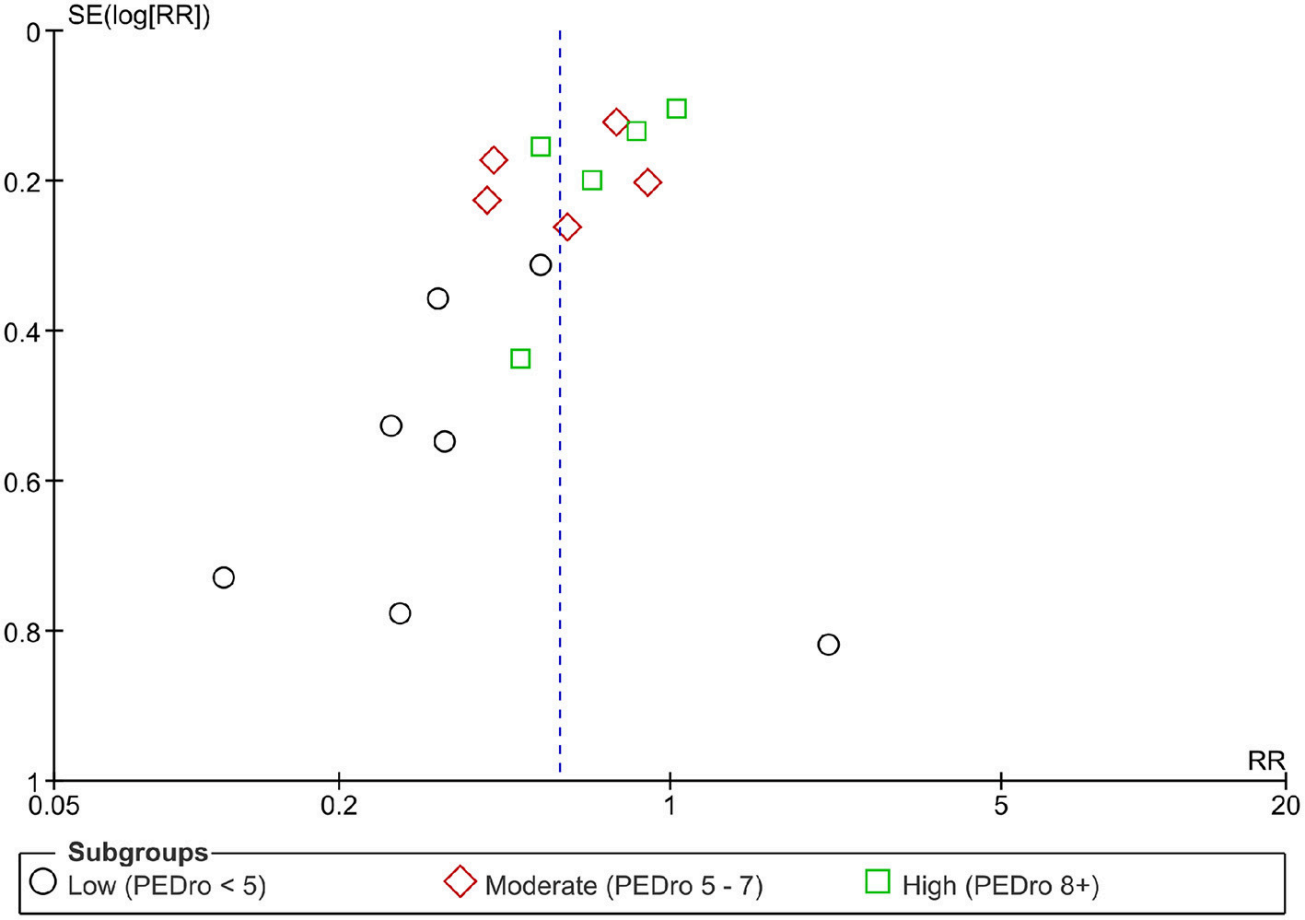
Funnel plot; SE, standard error, RR, relative risk.

Sensitivity analyses (Table 3 and Supplementary Material) revealed that the study design (randomized vs. non-randomized) had little impact on the effect estimate (*I*^2^ = 0%; *Q* = 0.14; *p* = 0.71). There was a significant difference between study quality subgroups (*I*^2^ = 66.1; *Q* = 5.90; *p* = 0.05), with higher effect estimates in studies with low internal validity (Pedro score <5; IRR = 0.37, 95% CI 0.23–0.60), compared to moderate (IRR = 0.60, 95% CI 0.44–0.82) and high (IRR = 0.74, 95% CI 0.56–0.98) PEDro scores. This was further supported by inspection of the funnel plot (Figure 2).

**Table 3:** Results of subgroup analysis.

**Moderator**	**Within-subgroup comparison**						**Heterogeneity (Chi**^**2**^ **test)**		
	**No. of effect sizes^a^**	**Total player exposures**	**IRR**	**95% CI**	***p*-value**	**Possible risk reduction, %**	***Q*-value**	***p*-value**	**I^2^**
**STUDY DESIGN AND QUALITY (SENSITIVITY ANALYSIS)**									
**Study design**									
Cluster RCTs	13	1,277,433	0.59	0.47–0.74	<0.001	41	49.81	<0.001	76
Cohort studies	4	147,324	0.52	0.27–1.00	0.050	48	4.4	0.22	32
				Test for subgroup differences			0.14	0.71	0
**Study quality**									
Low (PEDro ≤ 4)	7	389,003	0.37	0.23–0.6	<0.001	63	9.3	0.1	46
Medium (PEDro 5–7)	5	577,713	0.6	0.44–0.82	0.002	40	14.41	0.006	72
High (PEDro ≥ 8)	5	458,041	0.74	0.56–0.98	0.040	26	14.72	0.005	73
				Test for subgroup differences			5.9	0.05^*^	66.1
**MODERATOR ANALYSIS (DOSE-RESPONSE)**									
**Neuromuscular training dosage**									
**Session duration**									
Low (10–15 min)	5	175,445	0.55	0.42–0.72	<0.001	45	4.34	0.36	8
Medium (20–30 min)	10	1,152,336	0.6	0.46–0.76	<0.001	40	44.56	<0.001	80
High (<30 min)	1						*Not estimable*		
				Test for subgroup differences			0.21	0.65	0
**Training frequency**									
1x/wk	3	408,770	0.76	0.53–1.10	0.140	24	12.71	0.002	84
2x/wk	6	695,605	0.5	0.29–0.86	0.010	50	16.96	0.005	71
3x/wk	5	157,332	0.4	0.31–0.53	<0.001	60	1.02	0.91	0
>3x/wk	1						*Not estimable*		
				Test for subgroup differences			7.69	0.02^*^	74
**Weekly training volume**									
Low (20–30 min)	6	500,196	0.67	0.51–0.90	0.007	33	17.83	0.003	72
Medium (30–60 min)	6	688,655	0.45	0.25–0.81	0.008	55	19.16	0.002	74
High (>60 min)	4	188,258	0.54	0.32–0.90	0.020	46	11.41	0.01	74
				Test for subgroup differences			1.7	0.43	0
**Intervention volume and period**									
**Total number of training sessions**									
Low (18–29 sessions)	2	83,681	0.48	0.27–0.85	0.010	52	0.67	0.41	0
Moderate (30–60 sessions)	8	1,055,386	0.57	0.41–0.79	0.001	43	42.17	<0.001	83
High (90+ sessions)	4	140,348	0.51	0.28–0.90	0.020	49	9.13	0.03	67
				Test for subgroup differences			0.29	0.87	0
**Intervention period**									
Short term (1.5–6 months)	6	324,742	0.57	0.41–0.79	0.001	43	11.21	0.05	55
Long term (7–12 months)	10	1,043,399	0.57	0.42–0.76	<0.001	43	42.21	<0.001	79
				Test for subgroup differences			0	0.95	0

* Significant subgroup difference;

a *Number of individual IRRs considered for each subgroup comparison; IRR, injury rate ratio; CI, confidence interval; RCT, randomized controlled trial; wk, week*.

### Meta-analysis results: overall effect of NMT

**Figure 3:**
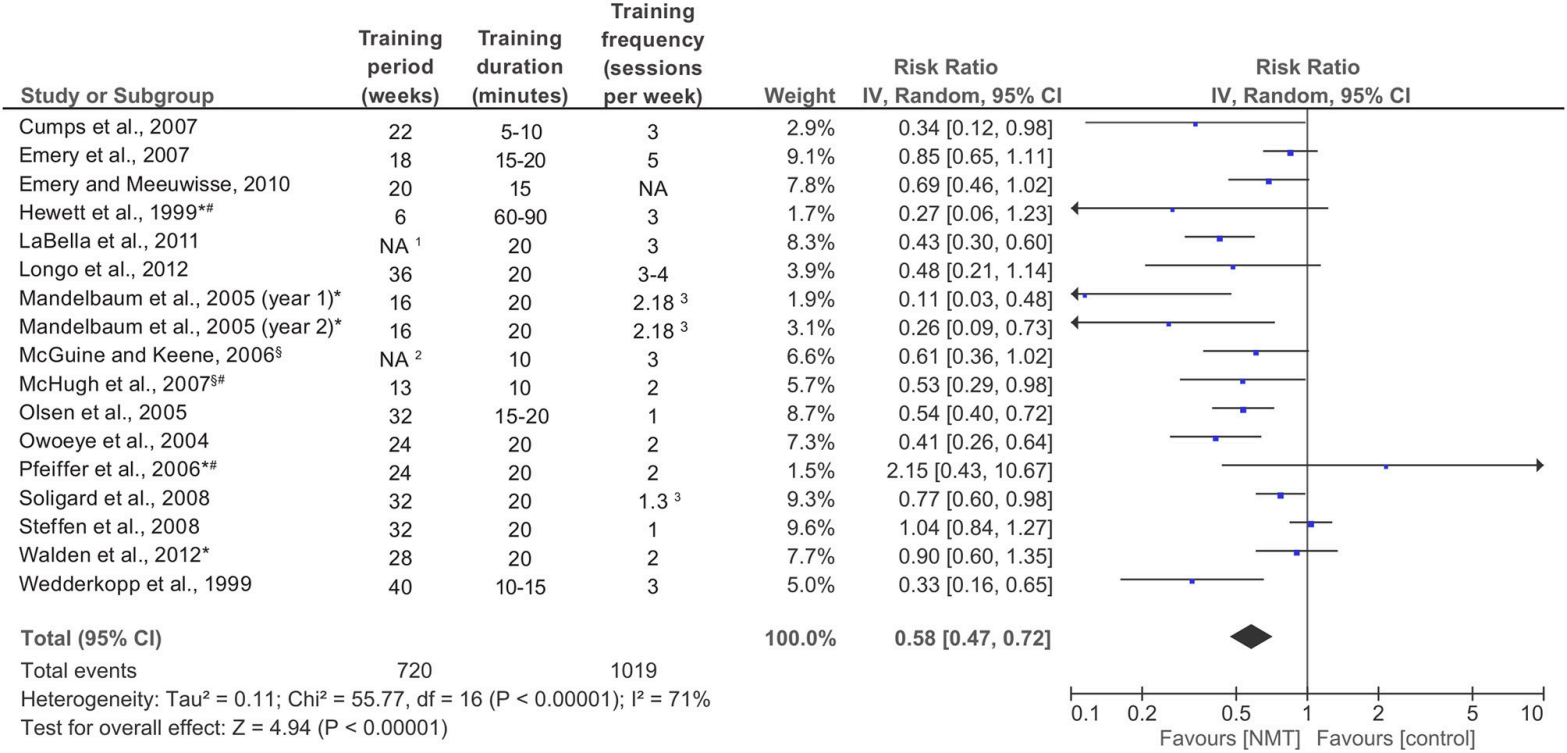
Forest plot with individual studies' injury risk ratios (IRRs) and the overall pooled IRR; IV, inverse variance; CI, confidence interval; NMT, neuromuscular training. ^*^Knee injury data only; ^§^Ankle injury data only; ^#^Cohort studies; ^1^15–18 (basketball); 9–12 (soccer); ^2^One highschool preseason + season (soccer, basketball); ^3^Data provided by the author.

A summary of the individual studies' IRRs and the meta-analysis is provided in Figure 3. Data was pooled from a total of 16 studies to establish the overall effect of NMT interventions, representing 1,417,730 player exposures, and 1,724 LE injuries. The pooled IRR was 0.58 (95% CI 0.47–0.72; *Z* = 4.94; *p* < 0.001), indicating a statistically significant LE injury risk reduction of 42%. A substantial amount of heterogeneity existed in individual studies' effect estimates (*I*^2^ = 71%; *Q* = 55.77; *p* < 0.001).

### Moderator analysis: dose-response relationships of NMT

**Figure 4:**
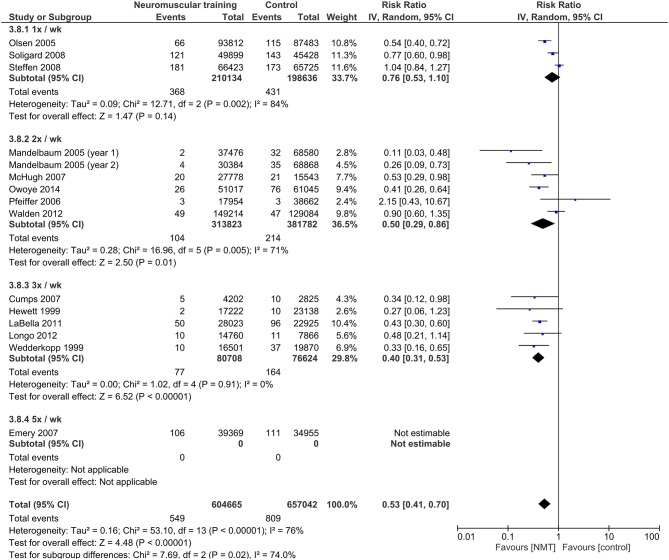
Subgroup analysis for the influence of NMT frequency on IRRs.

**Figure 5:**
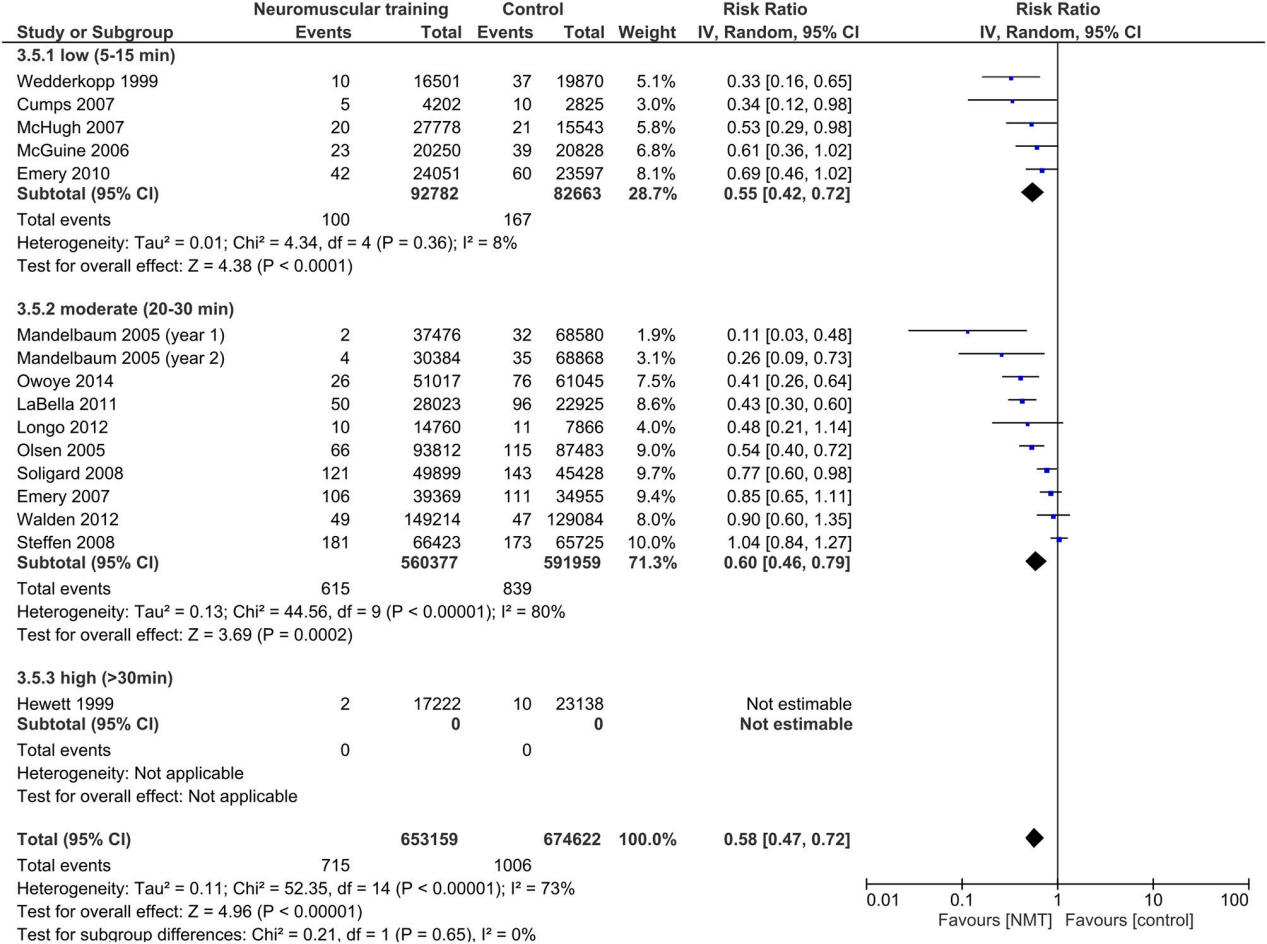
Subgroup analysis for the influence of NMT session duration on IRRs.

**Figure 6:**
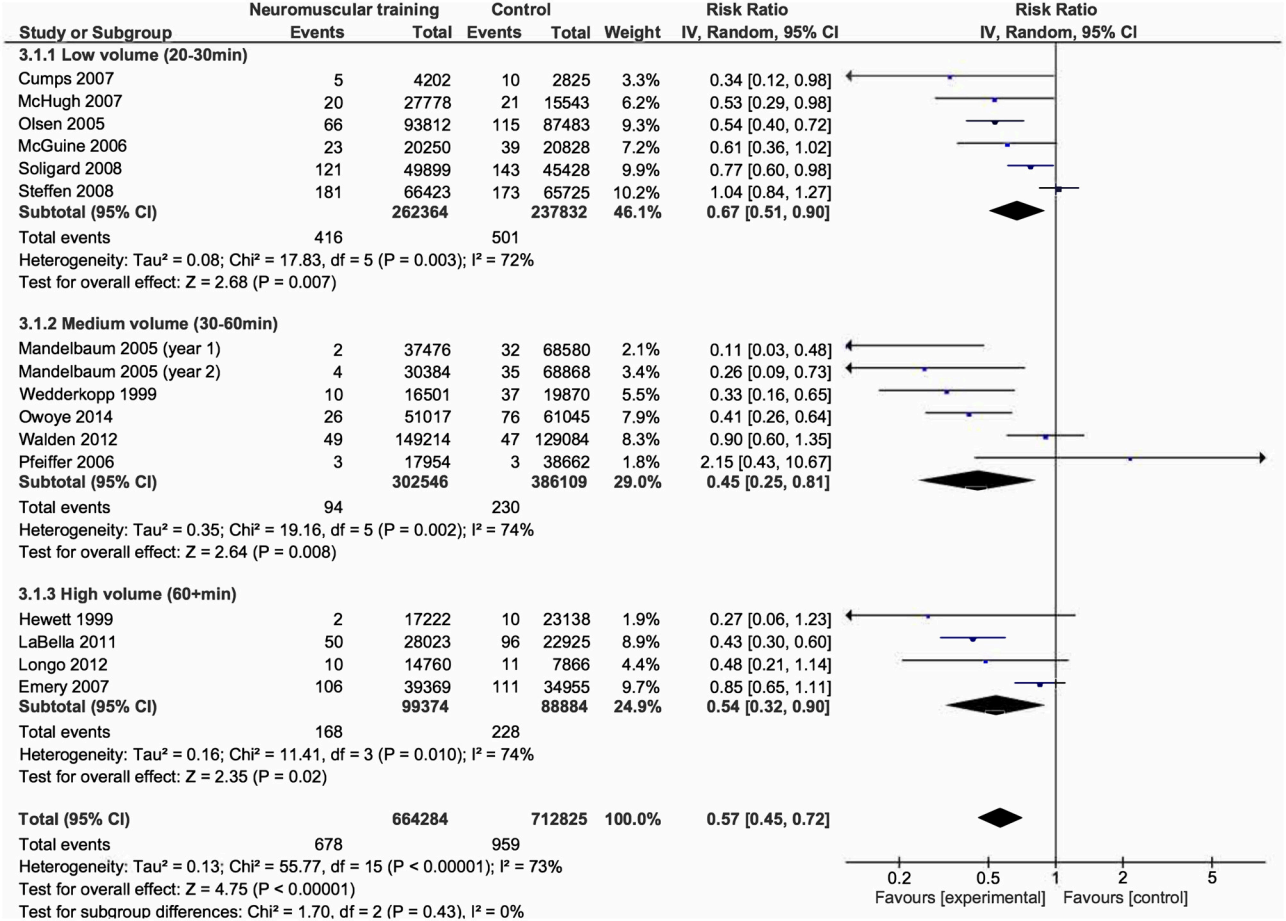
Subgroup analysis for the influence of weekly NMT volume on IRRs.

**Figure 7:**
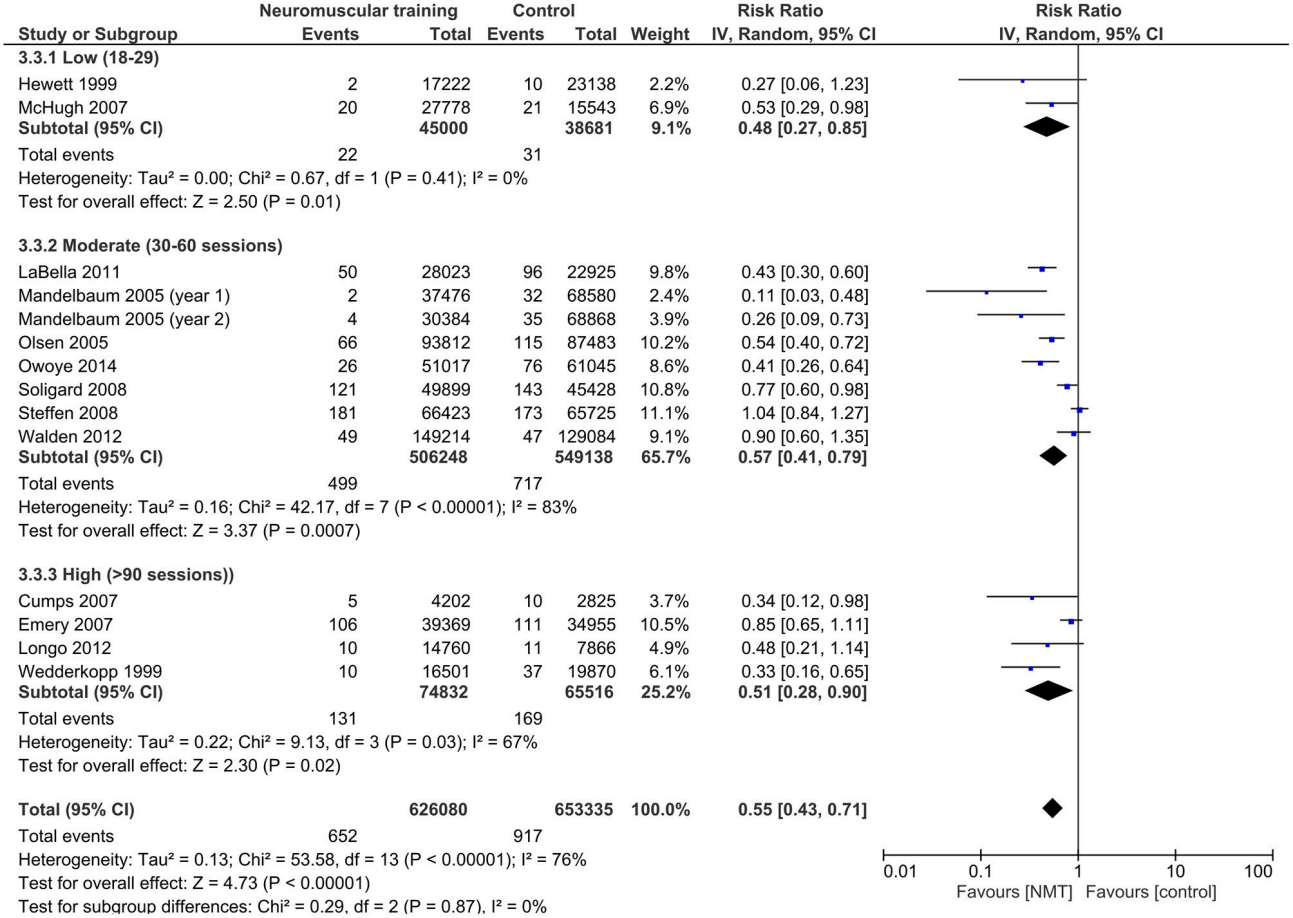
Subgroup analysis for the influence of the number of NMT sessions on IRRs.

**Figure 8:**
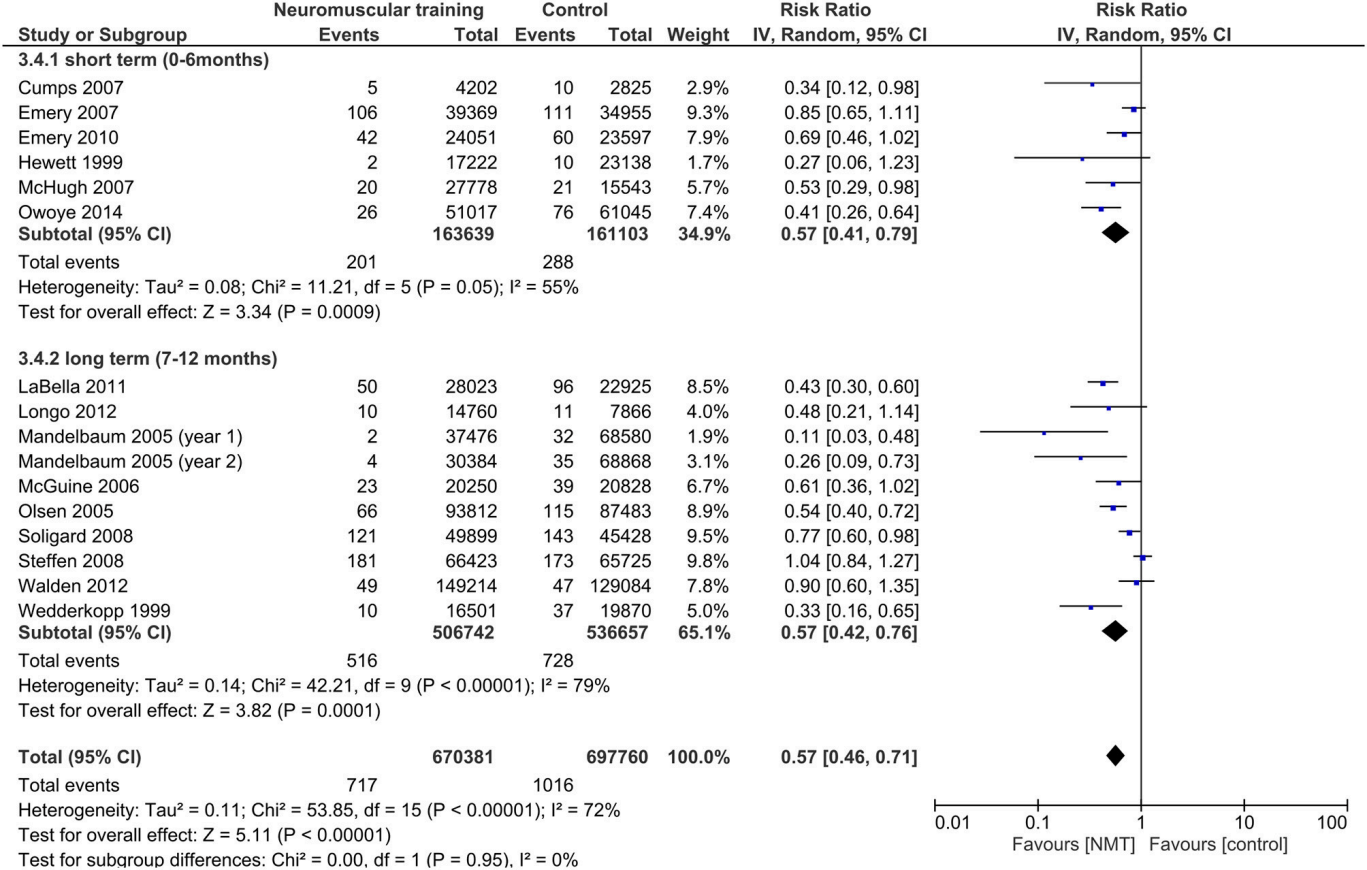
Subgroup analysis for the influence of NMT intervention period on IRRs.

Results from the moderator analyses are provided in Table 3 and Figures 4–**8**. We found a significant heterogeneity between *training frequency* subgroups (*I*^2^ = 74.0; *Q* = 7.69; *p* = 0.02), indicating differences in subgroups' pooled effect estimates (Figure 4). IRRs in trials with training frequencies of two (IRR = 0.5; 95%CI 0.29–0.86) or three times (IRR = 0.40; 95%CI 0.31–0.53) per week were lower (indicating higher risk reduction) compared to frequencies of once a week (IRR = 0.76; 95%CI 0.53–1.10). Programs with low (IRR = 0.55; 95%CI 0.42–0.72) NMT *session duration* produced effects comparable to those with medium session duration (IRR = 0.60; 95%CI 0.46–0.76; Figure 5). Further, a *weekly training volume* of more than 30 min tended to be more effective (30–60 min: IRR = 0.45; 95%CI 0.25–0.81; >60 min: IRR = 0.54; 95%CI 0.32–0.90) compared to lower volumes (20–30 min: IRR = 0.67; 95%CI 0.51–0.90; Figure 6).

Little differences existed between effect estimates of studies with moderate (IRR = 0.57; 95%CI 0.41–0.79) or high (IRR = 0.51, 95%CI 0.28–0.90) *total number of training sessions*, and studies with a low number of total sessions tended to have lower IRRs (IRR = 0.48; 95%CI 0.27–0.85; Figure 7). The *intervention period* had a negligible effect on pooled IRRs (0–6 months: IRR = 0.57, 95%CI 0.41–0.79; 7–12 months: IRR = 0.57, 95%CI 0.42–0.76; Figure 8).

## Discussion

The main aim of this meta-analysis was to identify the optimal training dose of NMT programs to reduce lower extremity injury risk in youth athletes. Overall, and consistent with previous reports, the included studies revealed a substantial preventive effect of NMT (IRR 0.58, 95% CI 0.47–0.72), with a 42% risk reduction for lower extremity injuries. Examination of dosage parameters indicated that the highest risk reductions were attained by NMT performed for two to three times per week, and a weekly training volume of 30–60 min. Consequently, injury prevention in youth athletes can be achieved with relatively modest training volumes. Pooled effects of training session durations indicated that short bouts of 10–15 min sessions may be sufficient to achieve this volume for a strong preventive impact. In terms of intervention period, preventive effects were already observed with fewer than 30 sessions and interestingly intervention periods of more than 6 months did not lead to an additional injury risk reduction. The results from the present meta-analysis should be interpreted with caution and considered a first step in understanding dose-response relationships, since comparisons are based on a relatively small number of studies with heterogeneous methodological quality.

### Duration, frequency, and volume

The results of this meta-analysis revealed that *training frequency* significantly affected the preventive effect of NMT. Programs with frequencies of two or three times a week demonstrated a substantially larger risk reduction compared to training once a week. Similar findings were demonstrated in a recent meta-analysis which investigated dose-response relationships of NMT to reduced ACL injury risk in young and adult female athletes (Sugimoto et al., 2014), which also suggested that the preventive effect increases with increasing numbers of weekly training sessions. Lesinski et al. (2015) reported that neuromuscular adaptations to balance training, a key component of NMT injury prevention programs, were high when conducted for two to three times a week. Thus, these training frequencies seem to be particularly effective for improving neuromuscular control, which has been proposed as a modifiable injury risk factor (Alentorn-Geli et al., 2009). From a practical point of view, this finding emphasizes the importance of regular implementation of NMT into an athlete's training routine. Since NMT programs, such as the “FIFA 11+” (Soligard et al., 2008), are typically designed as warm-up programs, this is easily achievable even in amateur level teams with less frequent training.

With respect to the optimal *NMT session duration*, our results indicate that session lengths of 10–15 min are sufficient to achieve a substantial risk reduction of 45%, with durations up to 30 min not appearing to have any additional effect. This is an important finding with respect to the practicability and feasibility of these programs, since it demonstrates that large preventive effects can be achieved with very short bouts of NMT. This makes exercise based injury prevention easily applicable for athletes and coaches, particularly in team sports settings where practice time is limited. While it is not possible to infer about the optimal timing within a practice session based on our meta-analysis, the integration of NMT bouts into athletes' warm-up routine was the most commonly chosen strategy. The efficacy of such NMT warm-up programs, such as the FIFA 11+, are well-established (Thorborg et al., 2017). Training effects may also be age-dependent, with Sugimoto et al. (2014) finding that session durations of more than 20 min were more effective for ACL injury prevention in both youth as well as adult female athletes. Thus, our results might point at a potential window of opportunity in young ages, where athletes might already benefit from short training bouts of <20 min, whereas longer sessions may be needed in older ages. This is further supported by a meta-analysis from Myer et al. (2013) who found an age effect for the effectiveness of NMT interventions, demonstrating a higher efficacy of NMT programs in young age groups. With respect to the underlying mechanisms, this finding could be explained by neuromuscular performance improvements, which have been shown to respond particularly well to short bouts of neuromuscular training sessions of 11–15 min (Lesinski et al., 2015). It is conceivable that the sensorimotor system in youth age has a greater potential for reorganization, which would consequently lead to more efficient and rapid adaptations during neuromuscular interventions. A second reason for the discrepancy between our findings and those of Sugimoto et al. (2014) may be related to the fact that they exclusively reviewed studies on female athletes. Thus, female athletes may respond better to training durations of more than 20 min, while young males might already adapt to shorter training stimuli. A previous meta-analysis by Rössler et al. (2014) demonstrated gender differences in the efficacy of exercise-based injury prevention programs, which would support this idea. However, it remains speculative based on the currently available data.

Our analysis revealed that a *weekly volume* of 30–60 min produced the highest injury risk reduction (IRR = 0.45; 95%CI 0.25–0.81), which equals two to three weekly sessions of 10–20 min duration. This finding confirms the aforementioned dosage effects for training frequency and duration, emphasizing the efficacy of short but frequent NMT sessions. In consequence, injury prevention is achievable with a modest volume of weekly training, which is a strong argument for the practicability of these intervention strategies. Coaches should aim at a regular incorporation of short NMT bouts in multiple practice sessions a week, which adds particular relevance to programs that can be incorporated in regular warm-up routines. Another strategy to ensure the required weekly volume is to have athletes perform additional sessions at home, since NMT programs are typically designed to require little space and equipment.

In summary, the results from this meta-analysis suggest that neuromuscular injury prevention programs should be conducted for at least two to three times a week, in short bouts of 10–15 min, ensuring a weekly volume of 30–60 min. This allows athletes and coaches to easily incorporate NMT contents into regular practice routines or in additional home training programs. Although, it is difficult to conclude on the optimal timing of NMT within a practice session based on the existing data, the majority of effective programs implement the training during athletes' warm-up.

### Intervention volume and period

The *total volume* and *period of NMT* interventions are additional important factors to consider. We found the largest preventive effects in studies with a low amount of sessions (IRR = 0.48; 95%CI 0.27–0.85). However, this category only contained two studies, which both demonstrated poor methodological quality (Hewett et al., 1999; McHugh et al., 2007). Thus, an overestimation of this effect is likely. More importantly, there was no difference in the preventive effects between studies with moderate (30–60) and high (>60) total numbers of training sessions. Our findings suggest that about 20–60 training sessions may already induce a considerable injury risk reduction provided a frequent incorporation into practice, which will then be sustained with further regular practice. In addition, analysis of the *total intervention period* revealed that studies with short-term interventions of 1.5–6 months demonstrated similar effects compared to studies with longer training periods of 7–12 months. A possible explanation for these findings might be the specific time course of training-induced neuromuscular adaptations: Neuromuscular performance improvements, including increased balance, muscular power, and strength, have recently been demonstrated after NMT injury prevention programs in youth athletes (Faude et al., 2017). These adaptations were consistently demonstrated within only a few weeks of training (Steffen et al., 2013; Zech et al., 2014; Rössler et al., 2016; Steib et al., 2016), and the meta-analysis by Lesinski et al. (2015) reported a peak after 11–12 weeks of training in healthy adults. Consequently, rapid initial adaptations in neuromuscular abilities might lead to the fast reductions of injury risk, potentially reaching a plateau after the first months of training. However, it is noteworthy that this comparison only considers the prescribed, but not the actual number of sessions completed by the athletes. This would obviously provide more valuable information (Stevenson et al., 2015), but was not available in most cases.

In summary, the present evidence indicates that substantial injury prevention can be expected with just a moderate amount of 20–60 training session, within a period of <6 months. From a practical point of view, this further emphasizes the value of incorporating NMT contents into regular practice at any time of the competitive season. Further, the data suggest that these early adaptations will be sustained with continuing training, which emphasize the value of a continuous incorporation of neuromuscular contents into the athletes' long-term training process.

### Limitations

We decided to include randomized and non-randomized as well as cohort designs in order to obtain more data for investigating dose-response relationships. A sensitivity analysis revealed that the study design (randomized vs. non-randomized) had no substantial effect on the studies' effect sizes, which is in accordance with the findings from a previous meta-analysis (Rössler et al., 2014). However, the methodological quality of included studies varied substantially, and one third of the included studies scored poorly on the PEDro scale. Inspection of the funnel plot as well as a sensitivity analysis of trial quality revealed a tendency for smaller sampled and low-quality studies to report greater risk reductions than larger trials with moderate to high quality. This may have affected some of our moderator analyses, particularly when subgroups contained only few studies. In addition, even where substantial differences for effect estimates existed between subgroups, confidence intervals showed considerable overlap. Thus, on the basis of the current existing data, results can only serve as a first step in understanding dose-response relationships and need to be treated with some caution.

Study populations were diverse with respect to the type of sport, the gender and competitive level of participants. Seven studies investigated female and two trials male athletes only. Participants' competitive level ranged from amateur to high school or elite levels. While Soomro et al. (2016) did not find differential effects of injury prevention programs on male and female youth athletes, Rössler et al. (2014) reported that girls benefit more substantially from exercise-based injury prevention programs compared to boys. Further, they demonstrated that studies including sub-elite level athletes tended to show greater risk reductions compared to studies on elite athletes (Rössler et al., 2014). Consequently, the heterogeneity of effects we observed in the meta-analysis is likely not only attributable to difference in the NMT programs' content and dosage, but may at least partly be explained by specific differences in study populations.

In addition, our meta-analytical approach cannot consider the influence of specific program contents and the interactions between individual training modalities (e.g., duration, frequency, intervention period). Hence, subgroup analyses for selected dosage parameters neglect differences between studies in other training modalities. This may also explain why there was still considerable heterogeneity in some of the subgroups investigated. Where possible, we made efforts to account for this aspect by combining several parameters (i.e., weekly volume). In addition, training intensity, which is difficult to specify in multi-component NMT programs, was not addressed due to the lack of reported data. Further, athletes' compliance data was not available for many studies, and thus, is not considered in the analyses. This however is an important aspect influencing the efficacy of injury prevention programs (Soligard et al., 2010; Hagglund et al., 2013). Lastly, a differentiation between the effects of NMT programs on different types of injuries (e.g., overuse vs. traumatic; ankle vs. knee injuries) would add additional value to the dose-response analysis. This, however, was not possible in the present study due to the little data available at present.

## Conclusion

In conclusion, this meta-analysis revealed that NMT performed in short bouts of 10–15 min, two to three times per week, with a weekly training volume of 30–60 min had the largest preventive effect for lower extremity injuries. These effects were already observed within 20–60 sessions and training periods of <6 months, and seem to be sustainable with continued regular practice. Consequently, our results emphasize the value of short NMT bouts, such as structured warm-up protocols or home-training programs, which foster the regular incorporation of NMT in athletes practice routines. The fact that even modest weekly training volumes achieve desirable effects should encourage coaches to implement NMT contents into their practice regimes. The conclusions from this meta-analysis mainly represent results from studies including youth athletes between the ages of 12 and 21 years, and inferences for injury prevention in children are not possible at present. Further, the data underlying the dose-response analyses are derived from a limited number of studies with partly low methodological quality, which reduces the strength of the present recommendations. Further studies are needed to better understand the optimal program contents and training dosage, and the underlying mechanisms. Studies directly comparing the effects of individual dosage components are lacking. More work in this field is important in order to better educate athletes and coaches with respect to designing effective injury prevention programs.

## Author contributions

SS had the idea, designed, and conducted this meta-analysis. ALR and SS developed the search strategy and conducted the systematic literature screening and quality assessment. ALR and SS were also responsible for data extraction and synthesis. ALR, KP and AZ assisted in designing the moderator analyses and were involved in discussing the results of the meta-analysis. SS wrote the manuscript draft. ALR, KP, and AZ critically revised the draft with respect to the intellectual content and approved the final version.

### Conflict of interest statement

The authors declare that the research was conducted in the absence of any commercial or financial relationships that could be construed as a potential conflict of interest.
