# Supplementary material for: Dose-Response Relationship of Neuromuscular Training for Injury Prevention in Youth Athletes: A Meta-Analysis
Source: Front Physiol. 2017 Nov 14;8:920. doi: 10.3389/fphys.2017.00920 (PMC5694483; doi:10.3389/fphys.2017.00920)
Supplement: Supplementary file 1 [file Table1.pdf]

Supplementary Material

DOSE-RESPONSE RELATIONSHIP OF NEUROMUSCULAR TRAINING FOR INJURY PREVENTION IN YOUTH ATHLETES: A META-ANALYSIS

Simon Steib[1*], Anna Lina Rahlf[2], Klaus Pfeifer[1], Astrid Zech[2]

*** Correspondence:** Simon Steib: simon.steib@fau.de

1 Detailed search strategy

PICOS search strategy

**P**opulation	youth OR preadolescen* OR pre-adolescen* OR adolescen* OR child*
**I**ntervention	"neuromuscular training" OR "sensorimotor training" OR "balance training" OR "proprioceptive training" OR "instability training" OR "perturbation training"<br>OR<br>"neuromuscular exercise*" OR "sensorimotor exercise*" OR "balance exercise*" OR "proprioceptive exercise*" OR "instability exercise*" OR "perturbation exercise*"<br>OR<br>"neuromuscular warm-up" OR "neuromuscular warmup"<br>OR<br>"neuromuscular program*" OR "sensorimotor program*" OR "balance program*" OR "proprioceptive program*" OR "instability program*" OR "perturbation program*"
**C**omparison	-
**O**utcome	Injur*
**S**tudy design	(cluster) RCT, quasi-experimental trials, cohort studies

Boolean terms

#1	youth
#2	preadolescen*
#3	pre-adolescen*
#4	adolescen*
#5	child*
**#6**	**#1 OR #2 OR #3 OR #4 OR #5**
#7	neuromuscular
#8	sensorimotor
#9	balance
#10	proprioceptive
#11	instability
#12	perturbation
**#13**	**#8 OR #9 OR #10 OR #11 OR #12**
#14	training
#15	exercise*
#16	program*
#17	warm-up
#18	warmup
**#19**	**#14 OR #15 OR #16 OR #17 OR #18**
**#20**	**#13 W/1 #19**
#21	injur*
**#22**	**#6 AND #20 AND #21**

2 Documentation of database search

Pubmed

Date: 12.01.2017

Filter: none

Search mode: All Fields

Result: 573

Search	Query	Items found
#4	Search (#1 AND #2 AND #3)	573
#3	Search injur*	1017779
#2	Search “neuromuscular training” OR “sensorimotor training” OR “balance training” OR “proprioceptive training” OR “instability training” OR “perturbation training” OR “neuromuscular exercise*” OR “sensorimotor exercise*” OR “balance exercise*” OR “proprioceptive exercise*” OR “instability exercise*” OR “perturbation exercise*” OR “neuromuscular warm-up” OR “neuromuscular warmup” OR “neuromuscular program*” OR “sensorimotor program*” OR “balance program*” OR “proprioceptive program*” OR “instability program*” OR “perturbation program*”	10668
#1	Search (youth OR preadolescen* OR pre-adolescen* OR adolescen* OR child*)	3260133

Scopus

Date: 12.01.2017

Filter: none

Search mode: Title/Abstract/Key Words

Result: 258

Search	Query	Items found
#4	(TITLE-ABS-KEY (youth OR preadolescen* OR pre-adolescen* OR adolescen* OR child) AND (TITLE-ABS-KEY(injur*)) AND (TITLE-ABS-KEY (TITLE-ABS-KEY ((training OR exercise* OR program* OR warm-up OR warmup) W/1 (neuromuscular OR sensorimotor OR balance OR proprioceptive OR instability OR perturbation*)))	258
#3	TITLE-ABS-KEY ((training OR exercise* OR program* OR warm-up OR warmup) W/1 (neuromuscular OR sensorimotor OR balance OR proprioceptive OR instability OR perturbation*))	6,918
#2	TITLE-ABS-KEY (injur*)	1,397,904

#1	TITLE-ABS-KEY (youth OR preadolescen* OR pre-adolescen* OR adolescen* OR child)	3,847,035

<u>SPORTDiscus</u>

Date: 12.01.2017

Filter: none

Search modes: Boolean/Phrase; All fields

<u>Result: 113</u>

Search	Query	Items found
S6	S1 AND S4 AND S5	113
S5	injur*	129,311
S4	(neuromuscular OR sensorimotor OR balance OR proprioceptive OR instability OR perturbation*) N1 (training OR exercise OR warm-up OR warmup OR program*)	2,243
S3	training OR exercise OR warm-up OR warmup OR program*	464,851
S2	neuromuscular OR sensorimotor OR balance OR proprioceptive OR instability OR perturbation*	38,496
S1	youth OR preadolescen* OR pre-adolescen* OR adolescen* OR child*	134,542

<u>The Cochrane Library (CENTRAL)</u>

Date: 12.01.2017

Filter: none

Search modes: Boolean/Phrase; All text

<u>Result: 113</u>

Search	Query	Items found
#1	youth OR preadolescen* OR pre-adolescen* OR adolescen* OR child* (Word variations have been searched)	184,142
#2	injur* (Word variations have been searched)	35,871
#3	(neuromuscular OR sensorimotor OR balance OR proprioceptive OR instability OR perturbation*) near/1 (training OR exercise OR warm-up OR warmup OR program*) (Word variations have been searched)	1,190
#4	#1 and #2 and #3	113

PEDro

Date: 11.01.2017

Filter: none

Search modes: Match all terms AND; Abstract & Title

Results: 237

**Search**	**Query**	**Items found**
#1	youth OR preadolescen* OR pre-adolescen* OR adolescen* OR child*, neuromuscular OR sensorimotor OR balance OR proprioceptive OR instability OR pertubation* OR training OR exercise OR warm-up OR warmup OR program* OR injur*	30.474
#2	neuromuscular AND injur*	121
#3	“neuromuscular training” AND injur*	41
#4	“proprioceptive training” AND injur*	14
#5	“balance training” AND injur*	38
#6	“perturbation training” AND injur*	3
#7	“neuromuscular exercise*” AND injur*	5
#8	“balance exercise*” AND injur*	6
#9	“proprioceptive exercise*” AND injur*	4
#10	“neuromuscular warm-up” AND injur*	4
#11	“balance program*” AND injur*	1
#12	#2 - #11	237

Only the records with positive results were listed in the table

3 Supplementary figures

Study or Subgroup	Neuromuscular training Events	Neuromuscular training Total	Control Events	Control Total	Weight	Risk Ratio IV, Random, 95% CI
**1.4.1 Low (PEDro < 5)**						
Cumps 2007	5	4202	10	2825	2.9%	0.34 [0.12, 0.98]
Hewett 1999	2	17222	10	23138	1.7%	0.27 [0.06, 1.23]
Mandelbaum 2005 (year 1)	2	37476	32	68580	1.9%	0.11 [0.03, 0.48]
Mandelbaum 2005 (year 2)	4	30384	35	68868	3.1%	0.26 [0.09, 0.73]
McHugh 2007	20	27778	21	15543	5.7%	0.53 [0.29, 0.98]
Pfeiffer 2006	3	17954	3	38662	1.5%	2.15 [0.43, 10.67]
Wedderkopp 1999	10	16501	37	19870	5.0%	0.33 [0.16, 0.65]
**Subtotal (95% CI)**		**151517**		**237486**	**21.7%**	**0.37 [0.23, 0.60]**
Total events	46		148			
Heterogeneity: Tau² = 0.14; Chi² = 9.37, df = 6 (P = 0.15); I² = 36%						
Test for overall effect: Z = 4.05 (P < 0.0001)						
**1.4.2 Moderate (PEDro 5 - 7)**						
LaBella 2011	50	28023	96	22925	8.3%	0.43 [0.30, 0.60]
McGuine 2006	23	20250	39	20828	6.6%	0.61 [0.36, 1.02]
Owoye 2014	26	51017	76	61045	7.3%	0.41 [0.26, 0.64]
Soligard 2008	121	49899	143	45428	9.3%	0.77 [0.60, 0.98]
Walden 2012	49	149214	47	129084	7.7%	0.90 [0.60, 1.35]
**Subtotal (95% CI)**		**298403**		**279310**	**39.2%**	**0.60 [0.44, 0.82]**
Total events	269		401			
Heterogeneity: Tau² = 0.09; Chi² = 14.41, df = 4 (P = 0.006); I² = 72%						
Test for overall effect: Z = 3.17 (P = 0.002)						
**1.4.3 High (PEDro 8+)**						
Emery 2007	106	39369	111	34955	9.1%	0.85 [0.65, 1.11]
Emery 2010	42	24051	60	23597	7.8%	0.69 [0.46, 1.02]
Longo 2012	10	14760	11	7866	3.9%	0.48 [0.21, 1.14]
Olsen 2005	66	93812	115	87483	8.7%	0.54 [0.40, 0.72]
Steffen 2008	181	66423	173	65725	9.6%	1.04 [0.84, 1.27]
**Subtotal (95% CI)**		**238415**		**219626**	**39.1%**	**0.74 [0.56, 0.98]**
Total events	405		470			
Heterogeneity: Tau² = 0.07; Chi² = 14.72, df = 4 (P = 0.005); I² = 73%						
Test for overall effect: Z = 2.08 (P = 0.04)						
**Total (95% CI)**		**688335**		**736422**	**100.0%**	**0.58 [0.47, 0.72]**
Total events	720		1019			
Heterogeneity: Tau² = 0.11; Chi² = 55.77, df = 16 (P < 0.00001); I² = 71%						
Test for overall effect: Z = 4.94 (P < 0.00001)						
Test for subgroup differences: Chi² = 5.90, df = 2 (P = 0.05), I² = 66.1%						

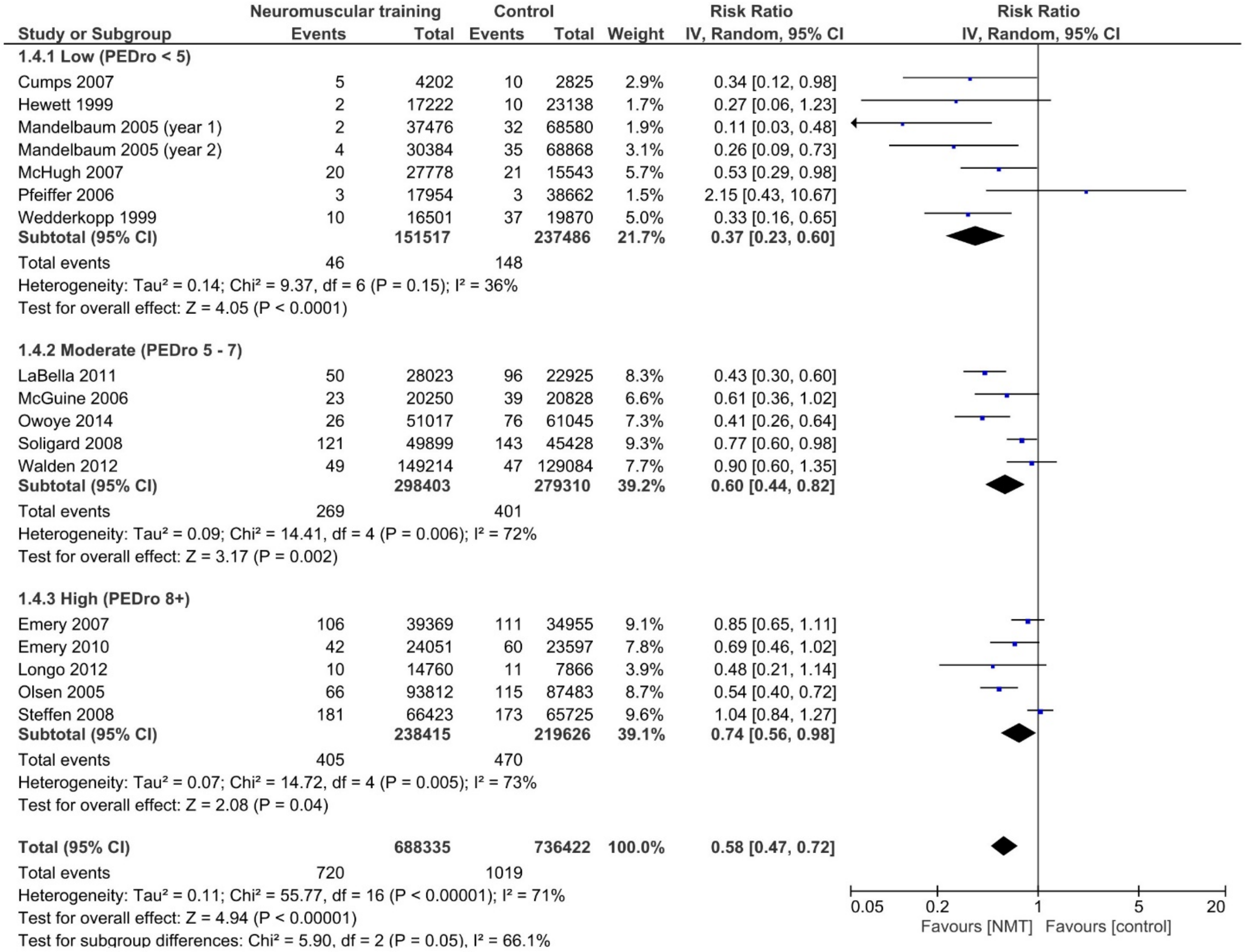

Supplementary Figure 1. Subgroup analysis for the influence of trial quality on IRRs

Study or Subgroup	Neuromuscular training Events	Neuromuscular training Total	Control Events	Control Total	Weight	Risk Ratio IV, Random, 95% CI
**1.3.1 RCTs**						
Emery 2007	106	39369	111	34955	9.1%	0.85 [0.65, 1.11]
Emery 2010	42	24051	60	23597	7.8%	0.69 [0.46, 1.02]
LaBella 2011	50	28023	96	22925	8.3%	0.43 [0.30, 0.60]
Longo 2012	10	14760	11	7866	3.9%	0.48 [0.21, 1.14]
Mandelbaum 2005 (year 1)	2	37476	32	68580	1.9%	0.11 [0.03, 0.48]
Mandelbaum 2005 (year 2)	4	30384	35	68868	3.1%	0.26 [0.09, 0.73]
McGuine 2006	23	20250	39	20828	6.6%	0.61 [0.36, 1.02]
Olsen 2005	66	93812	115	87483	8.7%	0.54 [0.40, 0.72]
Owoye 2014	26	51017	76	61045	7.3%	0.41 [0.26, 0.64]
Soligard 2008	121	49899	143	45428	9.3%	0.77 [0.60, 0.98]
Steffen 2008	181	66423	173	65725	9.6%	1.04 [0.84, 1.27]
Walden 2012	49	149214	47	129084	7.7%	0.90 [0.60, 1.35]
Wedderkopp 1999	10	16501	37	19870	5.0%	0.33 [0.16, 0.65]
**Subtotal (95% CI)**		**621179**		**656254**	**88.2%**	**0.59 [0.47, 0.74]**
Total events	690		975			
Heterogeneity: Tau² = 0.11; Chi² = 49.81, df = 12 (P < 0.00001); I² = 76%						
Test for overall effect: Z = 4.50 (P < 0.00001)						
**1.3.2 Cohort studies**						
Cumps 2007	5	4202	10	2825	2.9%	0.34 [0.12, 0.98]
Hewett 1999	2	17222	10	23138	1.7%	0.27 [0.06, 1.23]
McHugh 2007	20	27778	21	15543	5.7%	0.53 [0.29, 0.98]
Pfeiffer 2006	3	17954	3	38662	1.5%	2.15 [0.43, 10.67]
**Subtotal (95% CI)**		**67156**		**80168**	**11.8%**	**0.52 [0.27, 1.00]**
Total events	30		44			
Heterogeneity: Tau² = 0.15; Chi² = 4.40, df = 3 (P = 0.22); I² = 32%						
Test for overall effect: Z = 1.96 (P = 0.05)						
**Total (95% CI)**		**688335**		**736422**	**100.0%**	**0.58 [0.47, 0.72]**
Total events	720		1019			
Heterogeneity: Tau² = 0.11; Chi² = 55.77, df = 16 (P < 0.00001); I² = 71%						
Test for overall effect: Z = 4.94 (P < 0.00001)						
Test for subgroup differences: Chi² = 0.14, df = 1 (P = 0.71), I² = 0%						

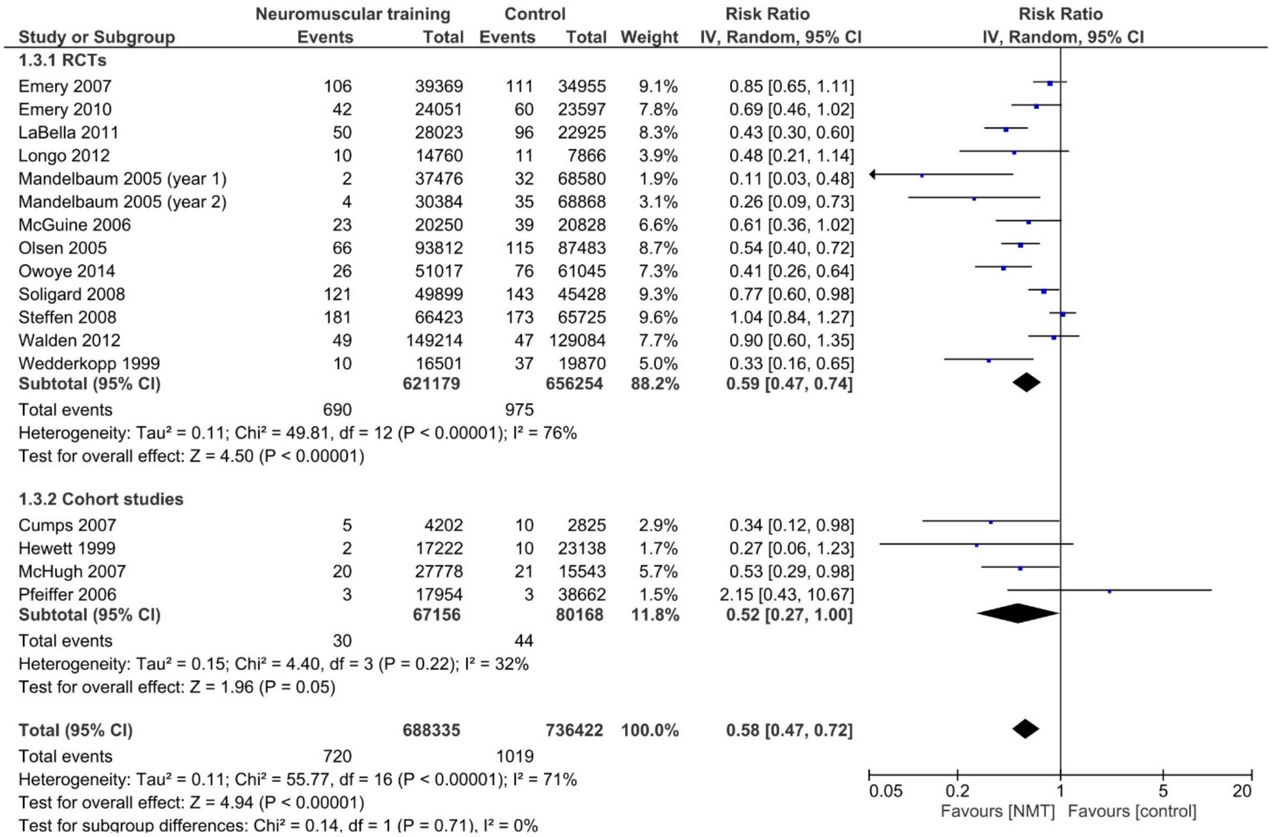

Supplementary Figure 2. Subgroup analysis for the influence of study type on IRRs
